# Make It Simple: (SR-A1+TLR7) Macrophage Targeted NANOarchaeosomes

**DOI:** 10.3389/fbioe.2018.00163

**Published:** 2018-11-06

**Authors:** Federico Leonel Parra, Ayelen Tatiana Caimi, Maria Julia Altube, Diego Esteban Cargnelutti, Mónica Elba Vermeulen, Marcelo Alexandre de Farias, Rodrigo Villares Portugal, Maria Jose Morilla, Eder Lilia Romero

**Affiliations:** ^1^Departamento de Ciencia y Tecnología, Nanomedicine Research & Development Center, Universidad Nacional de Quilmes, Bernal, Argentina; ^2^Centro Científico y Tecnológico de Mendoza, Instituto de Medicina y Biología Experimental de Cuyo, Consejo Nacional de Investigaciones Científicas y Técnicas, Mendoza, Argentina; ^3^Instituto de Medicina Experimental—Consejo Nacional de Investigaciones Científicas y Técnicas, Ciudad Autónoma de Buenos Aires, Argentina; ^4^Brazilian Nanotechnology National Laboratory, CNPEM, Campinas, Brazil

**Keywords:** archaeolipids, scavenger receptor, toll like receptor, endocytic internalization, interleukin 6

## Abstract

Hyperhalophilic archaebacteria exclusively produce sn2,3 diphytanylglycerol diether archaeolipids, unique structures absent in bacteria and eukaryotes. Nanovesicles made of archaeolipids known as nanoarchaeosomes (nanoARC), possess highly stable bilayers, some of them displaying specific targeting ability. Here we hypothesize that nanoARC made from *Halorubrum tebenquichense* archaebacteria, may constitute efficient carriers for the TLR7 agonist imiquimod (IMQ). NanoARC-IMQ takes advantage of the intense interaction between IMQ and the highly disordered, poorly fluid branched archaeolipid bilayers, rich in archaeol analog of methyl ester of phosphatidylglycerophosphate (PGP-Me), a natural ligand of scavenger receptor A1 (SR-A1). This approach lacks complex manufacture steps required for bilayers labeling, enabling future analytical characterization, batch reproducibility, and adaptation to higher scale production. SR-A1 mediated internalization of particulate material is mostly targeted to macrophages and is extensive because it is not submitted to a negative feedback. A massive and selective intracellular delivery of IMQ may concentrate its effect specifically into the endosomes, where the TLR7 is expressed, magnifying its immunogenicity, at the same time reducing its systemic bioavailability, and therefore it's *in vivo* adverse effects. NanoARC-IMQ (600–900 nm diameter oligolamellar vesicles of ~−43 mV Z potential) were heavily loaded with IMQ at ~44 μg IMQ/mg phospholipids [~20 folds higher than the non-SR-A1 ligand soyPC liposomes loaded with IMQ (LIPO-IMQ)]. *In vitro*, nanoARC-IMQ induced higher TNF-α and IL-6 secretion by J774A1 macrophages compared to same dose of IMQ and same lipid dose of LIPO-IMQ. *In vivo*, 3 subcutaneous doses of nanoARC-IMQ+ 10 μg total leishmania antigens (TLA) at 50 μg IMQ per Balb/C mice, induced more pronounced DTH response, accompanied by a nearly 2 orders higher antigen-specific systemic IgG titers than IMQ+TLA and LIPO-IMQ. The isotype ratio of nanoARC-IMQ+TLA remained ~0.5 indicating, the same as IMQ+TLA, a Th2 biased response distinguished by a pronounced increase in antibody titers, without negative effects on splenocytes lymphoproliferation, with a potential CD8+LT induction 10 days after the last dose. Overall, this first approach showed that highly SR-A1 mediated internalization of heavily loaded nanoARC-IMQ, magnified the effect of IMQ on TLR7 expressing macrophages, leading to a more intense *in vivo* immune response.

## Introduction

Archaebacterias are a unique and appealing source of new biomaterials. The diphytanylglycerol diether nature of archaeolipids, displaying a stereoisomerism different from that of lipids produced by bacteria and eukaryotes is responsible for the peculiar “membrane anomaly” described for Archaebacterias bilayers (Eme et al., 2017). In water media, polar archaeolipids form archaeosomes or nanoarchaeosomes (nanoARC), micrometer or submicrometer mean sized nanovesicles respectively, that chemical and biophysically differ from ordinary liposomes. In particular, archaeosomes prepared with polar archaeolipids extracted from *Halorubrum tebenquichense* contain ligands of SR-A1.

In this work we hypothesize that nanoARC prepared with lipids extracted from *Halorubrum tebenquichense* may constitute carriers that solve the problem of formulating the TLR7 ligand imiquimod (IMQ). IMQ is an immune response modifier, approved by the FDA for the treatment of actinic keratosis, superficial basal-cell carcinoma lesions, besides of various off-label uses on precancerous and cancerous skin lesions (Papadavid et al., 2007). Its use in preclinical transcutaneous immunization is limited however, since in the presence of a cytotoxic T lymphocyte (CTL) epitope, the elicited immune response decays within a few days, requiring co-stimulation via CD40 for instance, to enhance the primary CTL response and effective formation of memory CTL (Warger et al., 2007). Aldara, the same as other preclinical particulate carriers for IMQ so far developed, such as solid lipid nanoparticles (Zhou et al., 2007), liposomes (Fox et al., 2014), nanogels (Stein et al., 2014), or emulsions (Lopez et al., 2017), release IMQ into the application site microenvironment upon structural degradation of the carrier particle (Chollet et al., 1999). The IMQ spreading beyond the application site has been identified as a source of systemic toxicity (Steinhagen et al., 2011; Mifsud et al., 2014). Here we propose to modify the pharmacodynamics of IMQ by loading it within nanoARC. We hypothesize that nanoARC, by being naturally targeted to SRA-1 expressing macrophages, would deliver the carried IMQ into the endo-lysosomal pathway and therefore concentrate its effect into a specific intracellular site. IMQ induces a moderate TLR7-independent inhibition on adenylyl cyclase activity, impairing a negative feedback mechanism that normally limits inflammatory reactions (Schön et al., 2006; Schön and Schön, 2007). The avoidance of this inhibition by SR-A1 targeted delivery of IMQ and the lack of isostearic acid responsible for additional TLR7-independent inflammatory effects (Walter et al., 2013), would lower the chances of systemic inflammation a potential toxicity (Heikkinen and Susitaival, 2011). Switching from a diffusion mediated cell entering mechanism for free IMQ, to an endocytic uptake of nanoARC carrying IMQ (nanoARC-IMQ) by SR-A1+ cells would lead to a massive delivery IMQ to the endo-lysosomal system, concentrating its activity only on SR-A1+ and TLR7 expressing cells. This would improve IMQ immunogenicity, reducing at the same time its systemic bioavailability, and therefore it's *in vivo* adverse effects. Formulating the weak base IMQ [(1-(2-methylpropyl)-1 -imidazo [4,5-c] quinolin-4-amine] however, results a challenging task (Gogoll et al., 2012). There are a few organic agents (Chollet et al., 1999) capable of efficiently dissolve IMQ, such as the isostearic acid (ISA), a relatively toxic constituent of the poorly stable commercial cream Aldara (Chollet et al., 1999). The unique structural features of archaeolipid bilayers may help to partition the IMQ, ruling out the need for ISA. To the best of our knowledge, this is the first approach where a ligand expressed on the surface of nanoARC is combined with an immunomodulator, to render double targeted nanoARC. The lack of complex manufacture steps needed to covalent labeling is an additional benefit that will facilitate analytical characterization, batch reproducibility and adaptation to higher scale production of such formulation (Korsmeyer, 2016; Landesman-Milo and Peer, 2016). Here we will prepare and structurally characterize nanoARC-IMQ and test its *in vitro* and *in vivo* immunomodulatory performance upon 3 subcutaneous doses mixed with model mucocutaneous leishmania parasite proteins in Balb/C mice.

## Materials and methods

### Materials

Soybean phosphatidylcholine (SPC, purity > 90%) was a gift from Lipoid (Ludwigshafen, Germany). Imiquimod (purity >98%) was a gift from Laboratorio Lazar (Buenos Aires, Argentina). MONTANIDE™ ISA 763 A VG was from Seppic (Puteaux, France). Ficoll was from GE Healthcare (Munich, Germany). Hypaque was from Winthrop Products (Buenos Aires, Argentina). Roswell Park Memorial Institute (RPMI) 1640 medium was from Gibco, Life Technologies (New York, USA). Antibiotic/antimycotic solution (penicillin 10,000 IU/mL, streptomycin sulfate 10 mg/mL, amphotericin B 25 μg/mL), glutamine, and trypsin/ethylenediamine tetraacetic acid were from PAA Laboratories GmbH (Pasching, Austria). Fetal bovine serum (FBS) was from Internegocios, Cordoba, Argentina. Concanavalin A (ConA), Phorbol 12-myristate 13-acetate (PMA), Laurdan, saponin, Carboxy fluorescein succimidil ester (CFSE), 3-(4,5-dimethylthiazol- 2-yl)-2,5-diphenyl tetrazolium bromide (MTT), and 2, 2′-azino-bis (3-ethylbenzthiazoline-6-sulphonic acid (ABTS) were from Sigma-Aldrich (San Diego, USA). The other reagents were analytic grade from Anedra, Research AG (Buenos Aires, Argentina).

### Archaebacteria growth, extraction, and characterization of total polar archaeolipids

*Halorubrum tebenquichense* archaeas, isolated from soil samples of Salina Chica, Península de Valdés, Chubut, Argentina were grown in 15 L of basal medium supplemented with yeast extract and glucose at 37°C in a 25 L home-made stainless-steel bioreactor. Cultures were monitored by absorbance at 660 nm and harvested after 96 h growth. Total lipids were extracted from biomass using the Bligh and Dyer method modified for extreme halophiles and the Total Polar Lipid (TPL) fraction was collected by precipitation from cold acetone (Kates and Kushwaha, 1995). Between 400 and 700 mg of TPL were isolated from each culture batch. The reproducibility of each TPL extract's composition was routinely screened by phosphate content (Böttcher and Pries, 1961), and electrospray ionization mass spectrometry (ESI-MS) as described in Higa et al. (2012).

### Preparation of total *Leishmania amazonensis* antigen

Total L. amazonensis antigen (TLA) was prepared as described in Cargnelutti et al. (2016). Briefly, log-phase promastigotes of L. (L.) amazonensis were harvested by centrifugation, washed three times with PBS and disrupted by six to eight cycles of freezing (−80°C) and thawing (56°C). Protein contents of the TLA were estimated by the bicinchoninic acid assay (Micro BCA™ Protein Assay Kit, Thermo Fisher Scientific Inc., Massachusetts, USA). The antigens were kept frozen at −70°C until use.

### Preparation of nanoarc-IMQ and LIPO-IMQ

Nanoarchaeosomes made of TPL from *Halorubrum tebenquichense* (nanoARC), nanoarchaeosomes loaded with IMQ (nanoARC-IMQ), liposomes made of SPC (LIPO) and liposomes loaded with IMQ (LIPO-IMQ) were prepared by the lipid film hydration method. Briefly, to prepare nanoARC and LIPO, 50 mg of TPL or SPC, respectively, in 0.5 ml chloroform: methanol 1:1 (v/v) were poured in 2 ml U bottom shaped Eppendorf tube. The lipid film was formed upon evaporation of the organic solvent under N2 flush. Then, the films were hydrated with 1 ml of 10 mM Tris-HCl buffer pH 7.4 with 0.9% w/w NaCl (Tris-HCl buffer).

To prepare nanoARC-IMQ and LIPO-IMQ, the lipid films were hydrated with 1 ml of 2 mg/ml IMQ in 100 mM lactic acid (LH) under stirring.

To remove non-incorporated IMQ, nanoARC-IMQ and LIPO-IMQ were washed 3 times by 15 min centrifugation at 35,000 g, followed by supernatant removal and pellet resuspension in identical volume of LH up to no IMQ was detected in the washing media. Then the pellets were resuspended in 1 ml Tris-HCl buffer. Finally, nanovesicles were sonicated 1 h in bath sonicator (80 W, 80 KHz). To assess for a potential IMQ release from nanovesicles caused by sonication, the IMQ/phospholipids ratio was measured in nanoARC-IMQ and LIPO-IMQ prepared as stated above, excepting that half of the final pellets were washed in Tris-HCl buffer and half in LH. The IMQ/PL ratio is accurately estimated in LH, where the IMQ remains soluble, not co-precipitating with the pellets. Instead, an over estimation of the IMQ/PL ratio may occur in pellets washed in Tris-HCL buffer, where insoluble IMQ released from bilayers may co-precipitate with the pellets. Phospholipids and IMQ in pellets and supernatants were quantified as described below. The IMQ/PL of each pellet was calculated before and after sonication.

To assess for a potential IMQ release from nanovesicles after dilution, 1 ml of freshly prepared nanoARC-IMQ and LIPO-IMQ were washed by centrifugation by 15 min at 35,000 g followed by supernatant removal and pellet resuspension in 900 μl Tris-HCl buffer at 6, 24, and 48 h. Phospholipids and IMQ in supernatants were quantified as described below.

### Characterization of nanovesicles

#### Phospholipids and imiquimod quantification

Phospholipids (PL) were quantified employing a Böttcher microassay (Böttcher and Pries, 1961).

IMQ was quantified by UV-vis spectroscopy at λ 305 nm upon complete disruption of nanovesicles in ethanol:chlorhydric acid 98:2 v/v.

The absorbance of the sample was compared to a standard curve prepared with IMQ dissolved in ethanol:chlorhydric acid 98:2 v/v. The standard curve was linear in the range 1.25–20 μg/ml IMQ with correlation coefficient of 0.998.

#### Size and Z potential of nanovesicles

Size and Zeta potential were determined by dynamic light scattering (DLS) and phase analysis light scattering (PALS), respectively, using a nanoZsizer apparatus (Malvern Instruments, Malvern, United Kingdom). Nanovesicles were diluted 1:20 in Tris-HCl buffer (50 μl of samples with 950 μl of Tris-HCl buffer) for size and Z potential determinations.

#### Laurdan generalized polarization (GP) and fluorescence anisotropy (FA) of nanovesicles

The order and fluidity of nanovesicles bilayers before and after centrifugation were assessed by determining GP and FA, respectively of Laurdan. Briefly, nanovesicles were labeled with Laurdan by mixing 10 μl of 120 mM Laurdan in methanol with a volume of nanovesicles sufficient to render a 1:20 mol:mol Laurdan:lipids ratio.

GP was calculated using the following equation:

(1) $$\begin{array}{r} GP=-I_{490}/I_{440}+I_{490} \end{array}$$

Where I_440_ and I_490_ are the fluorescence intensities at λ_em_ = 440 nm and λ_em_ = 490 nm, respectively and obtained from the spectra between 400 and 520 nm at λ_ex_ = 364 nm (Slit_ex_: 5.0 nm and Slit_em_: 10.0 nm. Scan Speed: 100 nm/min).

FA was calculated by the fluorimeter software according to the following equation:

(2) $$\begin{array}{r} FA=\left(I_{0}-GI_{90}\right)/\left(I_{0}+2GI_{90}\right) \end{array}$$

Where I_0_ and I_90_ are the fluorescence intensities at λ_em_ = 440 nm with λ_ex_ = 364 nm and the excitation polarizer oriented at 0 and 90°, respectively. The correction factor (G) was obtained from the ratio of emission intensity at 0 and 90° with the excitation polarizer oriented at 90° (after subtraction of scattered light).

GP and FA measurements were carried out with a fluorescence spectrometer *LS 55, PerkinElmer*.

#### Cryo-transmission electron microscopy

Samples were prepared in a controlled environment vitrification system, equilibrated at 22°C and analyzed using a TALOS F200C (Thermo Fischer Scientific, USA) electron microscope operating at 200 kV. Images were acquired using a CMOS camera Ceta 16M 4k x 4k pixels (Thermo Fischer Scientific, USA). Images were not processed after acquisition. Sample preparation and data acquisition were performed at the Electron Microscopy Laboratory (LME)/Brazilian Nanotechnology National Laboratory (LNNano).

### Cytotoxicity and TNF-α and IL-6 induction on J774A1 macrophages

Immortalized murine Balb/C macrophages J774A.1 (ATCC® TIB-67™) were kindly supplied by Dr Erina Petrera (Facultad de Ciencias Exactas y Naturales Universidad Nacional de Buenos Aires, Argentina). The cells were maintained in RMPI 1640 supplemented with 10% FBS, 100 U/ml penicillin, 100 μg/ml streptomycin and 2 mM L-glutamine (complete RMPI medium) in a humidified atmosphere of 5% CO2 at 37°C.

The viability of J774A.1 cells upon 24 h incubation with 5, 0.5, 0.25, and 0.025 μg IMQ/ml (IMQ1,2,3, and 4, respectively), 100 and 10 μg PL/ml nanoARC (nanoARC1 and nanoARC2), 100 and 10 μg PL/ml nanoARC-IMQ (nanoARC-IMQ1 and nanoARC-IMQ2), 100 and 10 μg PL/ml LIPO (LIPO3 and LIPO4), and 100 and 10 μg PL/ml (LIPO-IMQ3 and LIPO-IMQ4) was measured by the MTT assay. The MTT test measures the activity of mitochondrial dehydrogenases and therefore reflects both cytostatic and cytotoxic effects.

To visualize potential morphological changes suffered by J774 A1 cells upon 24 h incubation with the above-mentioned samples, a series of bright field microscopies were taken. To that aim, 150,000 cells were seeded in 300 μl RPMI culture media with 5% FBS per well in a 24 wells plaque. The cells were cultured in CO2 at 37°C along 24 h. Afterwards the culture media was extracted, and samples diluted in culture media were added and cultured along 24 h in identical conditions. Before each picture was taken, the supernatant was removed and two washes with non-sterile PBS were performed. The bright field microscopy images at 20X magnification were taken employing a Cytation™ 5 Cell Imaging Multi-Mode Reader (BioTek Instruments, Inc., Winooski, VT, USA) equipment. The assay was performed by triplicate, employing 3 independent batches; two pictures per well were taken and the more representative images were selected.

The release of TNF-α and IL-6 in supernatants was evaluated after incubation with nanovesicles on J774A.1. LPS at 1 μg/ml was used as positive control. Mouse TNF-α and IL-6 in supernatants, were measured by enzyme-linked immunosorbent assay (ELISA) (BD, OptEIA, BD Biosciences, CA, USA), following the manufacturer instructions.

### Immunization

#### Animals and immunization schemes

6–8 weeks female BALB/cAnN mice were obtained from Centro Atómico de Ezeiza; Comisión Nacional de Energía Atómica (CNEA). Five mice randomly selected were distributed per box and housed in a ventilated room under controlled conditions, at constant temperature of 22°C, 12/12 h light/darkness cycles and food and water *ad libitum*. This study was carried out in accordance with the principles of the Institutional Committee of Care and Use of Laboratory Animals (CICUAL) of the National Quilmes University, that follows the International guiding principles for biomedical research involving animals of the Council for International Organization of Medical Sciences and the International Council for Laboratory Animal Science (CIOMS-ICLAS). The protocol was approved by CICUAL of the National Quilmes University.

A first series of subcutaneous immunization employing five mice per group was performed on days 0, 14, and 28. Immunization were carried out with 10 μg TLA physically mixed with MONTANIDE™ ISA 763 A VG (ISA 763) (prepared as a water in oil emulsion at adjuvant:antigen 7:3 v:v) according to the manufacturer instructions (Technical Bulletin, Seppic, Puteaux, France), or mixed with 50 μg IMQ either free (a 1:10 Tris-HCl buffer IMQ dilution from a 10 mg/ml IMQ stock solution in 100 mM LH) or from the following formulations: nanoARC-IMQ, ARC+IMQ, LIPO-IMQ, LIPO+IMQ (where nanoARC-IMQ+TLA = IMQ loaded in nanoARC mixed with TLA; ARC+IMQ+TLA = a mixture of IMQ, nanoARC and TLA; LIPO-IMQ+TLA = IMQ loaded in liposomes mixed with TLA and IMQ+LIPO+TLA = a mixture of IMQ, liposomes, and TLA).

A second series of subcutaneous immunization was performed following the same time schedule, this time with nanoARC-IMQ+TLA, nanoARC+IMQ+TLA, LIPO-IMQ+TLA, ISA763+TLA, IMQ+TLA, and TLA.

#### DTH

Delayed type hypersensibility reaction (DTH) was tested 10 days after the last immunization (day 38), according to Luo and Dorf (2003) with some modifications. Briefly, 10 μg of TLA diluted in 30 μl of PBS was injected subcutaneously into the dorsal left footpad of each mice. After 24 h, right and left footpad thickness was measured with a manual caliper. The swelling response was calculated subtracting the right footpad thickness (baseline) from that of the left footpad (injected with TLA).

#### Humoral response

Blood was collected from mice tail veins at day 38 and IgG, IgG1, and IgG2a antibody responses were measured by enzyme-linked immunosorbent assay (ELISA) in serum samples. Briefly, high-affinity 96 wells plates were coated overnight at 4°C with 100 μl of TLA (3 μg/well) in carbonate-bicarbonate buffer (pH 9.6). After washing three times with PBS with 0.05% Tween 80 (PBST), non-specific sites were blocked during 1 h at 37°C with 0.1% BSA in PBST (PBST-BSA). After another series of washes with PBST, 100 μl of three-fold dilutions of serum in PBST-BSA were incubated during 1 h at 37°C. Then, the plate was washed three times with PBST and incubated during 1 h at 37°C with horseradish peroxidase-conjugated goat anti-mouse total IgG (Millipore-Chemicon International, Temecula, USA) diluted 1:5,000 in PBST-BSA. To determine the antibody isotyping, horseradish peroxidase-conjugated rat anti-mouse IgG1 or IgG2a revealing antisera (PharMingen, San Diego, USA), diluted 1:5,000, were used. The plates were further washed and incubated with ABTS for 15 min at room temperature (20°–25°C) in the dark. The absorbance was measured at 405 nm using a microplate reader. Antibody titers were represented as end-point dilutions exhibiting an optical density of 0.3 units above background.

#### IFN-γ in splenocytes supernatant, intracellular IL-10 and IL-12 cytokines

At day 38, the spleens of mice immunized with nanoARC-IMQ+TLA, nanoARC+TLA, ISA 763+TLA were removed aseptically and splenocytes were obtained using a Ficoll-Hypaque gradient, as described by Böyum (1968). Briefly, spleens were disaggregated on a metal mesh employing non-supplemented RPMI media. The splenocytes were isolated by centrifuging 30 min at 500 g in a Ficoll-Hypaque gradient (1.098 g/ml density) in a Gelec G144d centrifuge. The resultant pellet was washed twice with RPMI and finally suspended in supplemented RPMI (10% SFB; 100 U/ml penicillin; 100 mg/ml streptomycin; 50 μM 2-mercaptoethanol). The cells were counted in a Neubauer chamber employing Türk dye and seeded in 96 wells U shape bottom plate at a density of 1 × 106 cells per well in a final volume of 200 μl. The cells were stimulated *in vitro* with TLA (3 μg/well) or concanavalin A (1 μg/well) as positive control. Non-stimulated cells were used as negative control. Supernatants were collected after 48 h incubation at 37°C in a humified chamber containing 5% CO2. The release of IFN-γ in cells supernatant was measured by ELISA (BD OptEIA™, BD Biosciences, San Jose, USA) following the manufacturer instructions. Absorbance measurements were carried out at 450 nm in a microplate reader.

To measure intracellular cytokines, cells were marked with FITC conjugated anti-MHCII(I-Ad) and fixed in 1% paraformaldehyde. Then, cells were permeated with 0.5% saponin in PBS and intracytoplasmic marking of IL-10 and IL-12 was done using PE conjugated antibodies (Biolegend, San Diego, USA). For this, cells were previously incubated during 4 h with Brefeldin A (Thermo Fisher Scientific, Waltham, USA) to inhibit cytokines liberation to external medium. Cytokine production analysis was done with a FACScan flow cytometer and CellQuest software. Results were shown as the percentage of positive cells for each marker.

#### Splenocytes surface markers

Splenocytes from immunized and from non-immunized mice were marked with fluorescent surface membrane monoclonal antibodies (FITC conjugated anti-CD8, anti-MHCII(I-Ad) and anti-CD11c; PerCP conjugated anti-CD4 and anti-B220; PE conjugated anti-CD11b) (BioLegend, San Diego, USA) according to Foster et al. (2007). Briefly, cell suspensions were incubated with the indicated antibodies, diluted in PBS at 4°C during 30 min and then washed with PBS and fixed in 1% paraformaldehyde. Cell surface markers analysis was done with a FACScan flow cytometer and CellQuest software (BD Bioscience, San Jose, USA). Results are shown as the percentage of positive cells for each marker.

#### Lymphocyte proliferation assay

Antigen specific cell response was also evaluated by a lymphocyte proliferation assay using 5 nM of CFSE, according to Lyons and Parish (1994). Sixty days post-immunization, splenocytes from immunized and from non-immunized mice were harvested. Spleen cells were incubated with CFSE during 30 min at 37°C and then washed twice with RPMI with 0.5% of FBS. Finally, cells were seeded at a density of 1 × 106 in 200 μl of complete RPMI and were stimulated with TLA (3 μg/well) or PMA (0.2 ng/well). Non-stimulated cells were used as negative control. Proliferating cell percentage was measured by FACScan flow cytometer at time 0 (basal), 24 and 48 h of incubation and data was analyzed with CellQuest software. Results were expressed as mean fluorescence intensity (MFI).

### Statistical analysis

Statistical analysis was performed by two-way analysis of variance (ANOVA) followed by Tukey's multiple comparisons or one-way ANOVA followed by Dunnet's test using Prism 6.0 Software (GraphPad, San Diego, California). *P* < 0.05 was considered statistically significant. ^*^p < 0.05; ^**^p < 0.01; ^***^p < 0.001; ^****^p < 0.0001; ns: represents not significant (*p* > 0.05).

## Results

### Nanonanovesicles structure

**Figure 1:**
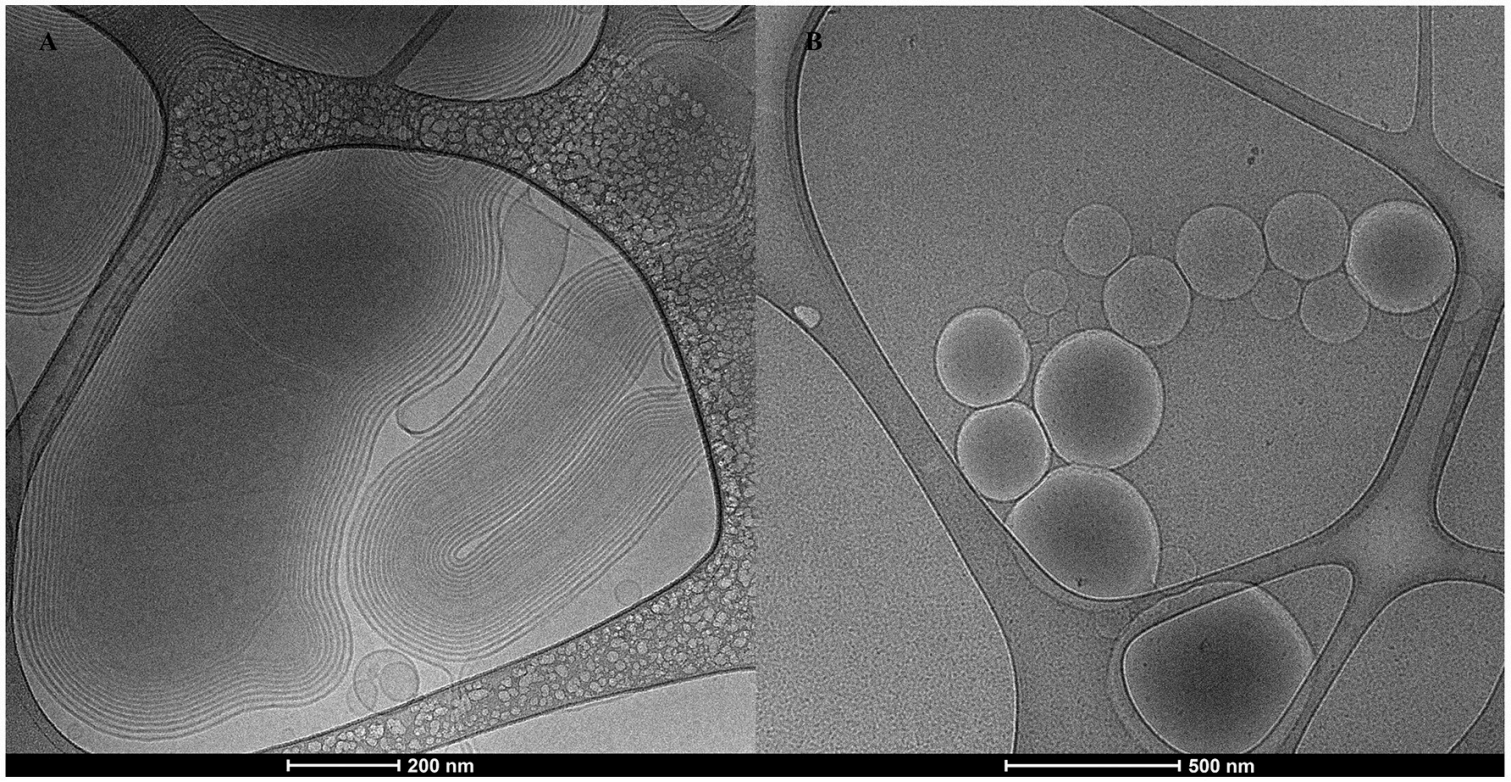
**(A)** Cryo-TEM image of nanoARC-IMQ+TLA, **(B)** Cryo-TEM image of LIPO-IMQ+TLA.

The Cryo-TEM images depicted in Figure 1A, showed nanoARC-IMQ as heterogeneous populations, consisting of 600–800 nm oligolamellar nanovesicles mixed with bigger sized multilamellar nanovesicles; in Figure 1B LIPO-IMQ are seen as oligolamellar vesicles having a dark material inside.

In coincidence with cryo-TEM images, DLS measurements in Table 1 showed highly polydisperse nanovesicles of diameters following the order: LIPO-IMQ > LIPO > nanoARC-IMQ > ARC. Because of its negative Z potential, no electrostatic association between TLA and nanovesicles occurred.

**Table 1:** Structural characterization of nanovesicles.

**Formulation**	**Mean diameter (nm)**	**PDI (mean ±SD)**	**Z potential (mV)**	**IMQ/lipid ratio (μg/mg)**
nanoARC	655 ± 271	0.62 ± 0.19	−42.6 ± 2.84	
nanoARC-IMQ	850 ± 300	0.73 ± 0.18	−42.9 ± 1.87	44 ± 10
LIPO	1630 ± 269	0.56 ± 0.16	−3.3 ± 0.14	
LIPO-IMQ	3614 ± 1787	0.73 ± 0.27	−5.4 ± 3.2	2.5 ± 1.5
TLA	6600 ± 1500	0.60 ± 0.20	−12 ± 0.5	

*Values are expressed as mean ± SD (n = 3 batches)*.

*PDI, Polydispersity Index; SD, standard deviation*.

**Figure 2:**
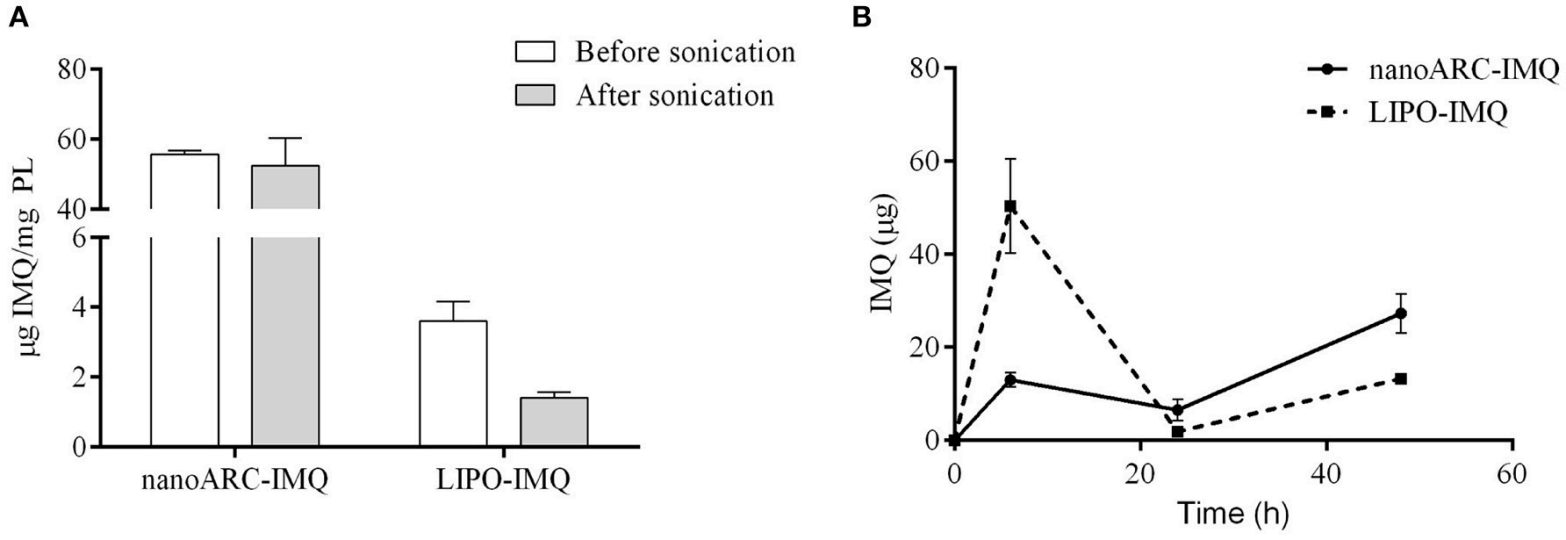
**(A)** IMQ/PL ratio of nanovesicles before and after sonication in lactic acid (LH), **(B)** released IMQ in supernatant.

As shown in Figure 2A, sonication reduced the IMQ/PL ratios from nanoARC-IMQ and LIPO-IMQ IMQ/PL. Notably, no differences were found between IMQ/PL ratio from nanoARC-IMQ measured in LH or Tris-HCl buffer washes, remaining almost invariant after sonication, decreasing by ≈15% to 44 μg IMQ/mg PL and resulting nearly 20-folds higher than that of LIPO-IMQ. This suggests that the IMQ was extensively partitioned in the nanoARC bilayer, and that it was not detached upon sonication. On the contrary the much lower IMQ/PL ratio of LIPO-IMQ was sensitive to sonication, decreasing by ≈65% in Tris-HCl buffer to 5 μg IMQ/mg PL [data not shown] and by ≈86% in LH to 2 μg IMQ/mg PL. In other words, the sonication induced the loss of nearly 90% of the IMQ in LIPO-IMQ while nearly half of quantified IMQ in Tris-HCl buffer was co-precipitated with the LIPO-IMQ pellet free IMQ. The encapsulation efficiency of IMQ was ≈38% in ARC-IMQ and ≈1% in LIPO-IMQ. As hypothesized by Fox et al. (2014), nanovesicles with a LH acid core would facilitate encapsulation of soluble IMQ while the bulk aqueous phase of nanovesicles remains close to a neutral pH, which is desirable to minimize injection pain (Fransson and Espander-Jansson, 1996). Here a LH solution of IMQ was used to hydrate the lipid films. However, despite of the reported low H+ permeability of ARC compared to that of liposomes (Yamauchi et al., 1993), upon centrifugation washes no acid media was detected within the vesicles (data not shown). The initial huge osmotic gradient, -in the order or several pH units- established across the nanovesicles bilayers along Tris-HCl buffer washes, may have induced swelling and further inner content leakage. Unless the IMQ was partitioned in bilayers, most of soluble IMQ would be removed upon leakage and washes (Figure 1B).

Figure 2B on the other hand, depicted the resultant of submitting the vesicles to 3 sequential washes in Tris-HCl buffer, aiming to assess a potential release of IMQ. Tough no significant phospholipids content was found in the supernatants (data not shown), different relative amounts of released IMQ were found instead: while LIPO-IMQ lost ~90% of its initial IMQ content, only ~10% of its initial IMQ content was lost from ARC-IMQ. The samples volume submitted to centrifugation was 1,000 μl and the pellet volume was ~100 μl. Hence, it was observed that ARC bilayers not only partitioned a higher amount of IMQ, but that the IMQ remained associated in a higher extent in front to a 1/10 folds dilution, than LIPO bilayers. This assay was aimed to simulate the IMQ release from each type of lipid matrix and represented a response to a potential dilution. *In vivo* however, 100 μl volume samples were injected in a virtual subcutaneous space, suffering a practically negligible dilution. Overall, from a structural point of view, ARC-IMQ would result better suited than LIPO-IMQ to provide intracellular delivery of IMQ, since a higher IMQ remained more strongly associated per mass of lipid matrix, favoring thus its endocytic uptake.

### Fluidity and order of bilayer nanovesicles

**Figure 3:**
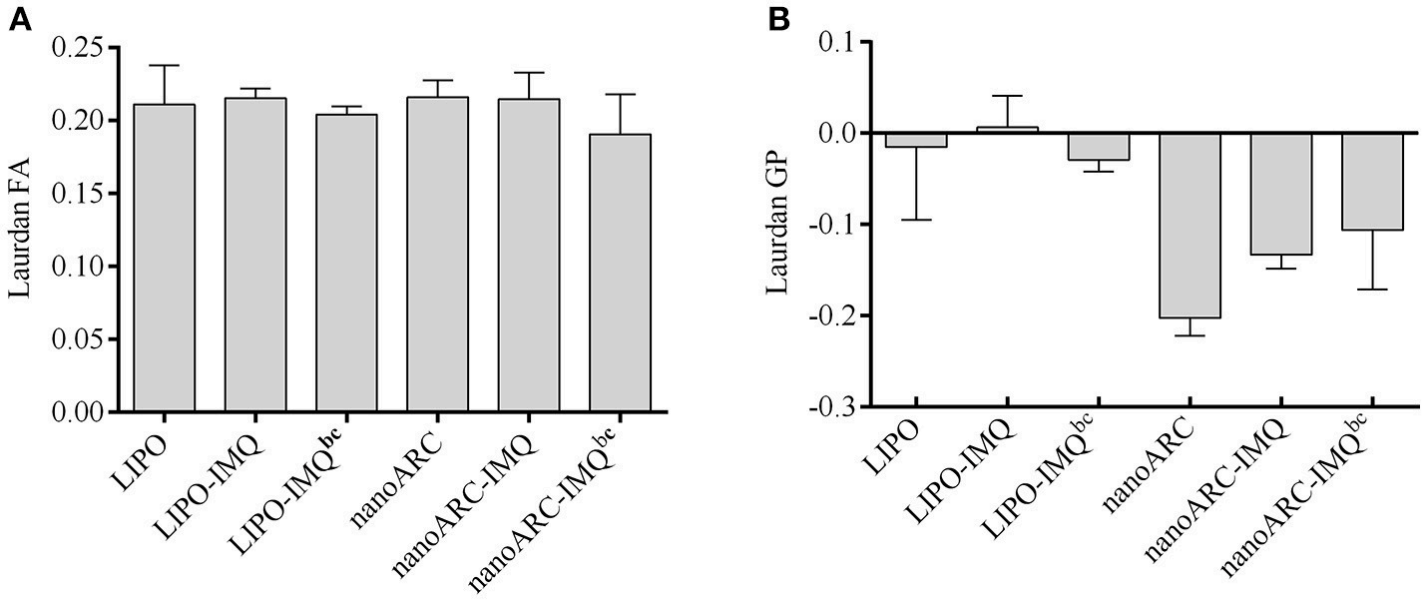
**(A)** Laurdan fluorescence anisotropy (FA) and **(B)** generalized polarization (GP) of nanovesicles; bc: before centrifugation. Values are expressed as mean ± SD (*n* = 3 batches).

The effect of IMQ on fluidity and order of nanoARC-IMQ and LIPO-IMQ bilayers was assessed according to Altube et al. (2017). To that aim, the fluorescent probe of membrane structure, sensitive to the solvent relaxation effect Laurdan was employed to determine generalized polarization (GP) and fluorescence anisotropy (FA), respectively. As depicted in Figure 3, IMQ did not modify nanovesicles bilayers fluidity that showed identical FA before and after IMQ loading. IMQ on the other hand, did not affect the order of LIPO bilayers, but deeply reduced the typical high disorder of ARC bilayers (Altube et al., 2017); the trend followed by the GP values was: nanoARC-IMQbc> nanoARC-IMQ (finished formulation) > ARC. The GP of the more ordered nanoARC-IMQbc bilayer was reduced after washing loosely trapped IMQ in nanoARC-IMQ; in other words, the order decreased as IMQ was removed. As shown in Table 1, trapped IMQ was not observed to reduce the negative Z potential of nanoARC-IMQ, despite of IMQ is positively charged at acid/neutral pH (Chollet et al., 1999). Our results suggested thus, that IMQ was partitioned modifying the order of the archaeolipid bilayer, without relevant electrostatic interaction between IMQ and the highly negative Z potential archaeolipid bilayers. Opposite to ARC, neither fluidity not order of LIPO bilayers were affected by IMQ, suggesting that IMQ was not trapped into LIPO bilayers but dissolved in minute amount into the aqueous space. This could account for the low IMQ/PL ratio, encapsulation efficiency, and structural sensitivity to sonication of LIPO-IMQ.

### Nanovesicles cytotoxicity and induction of TNF-α and IL-6 on J774A.1 macrophages

**Figure 4:**
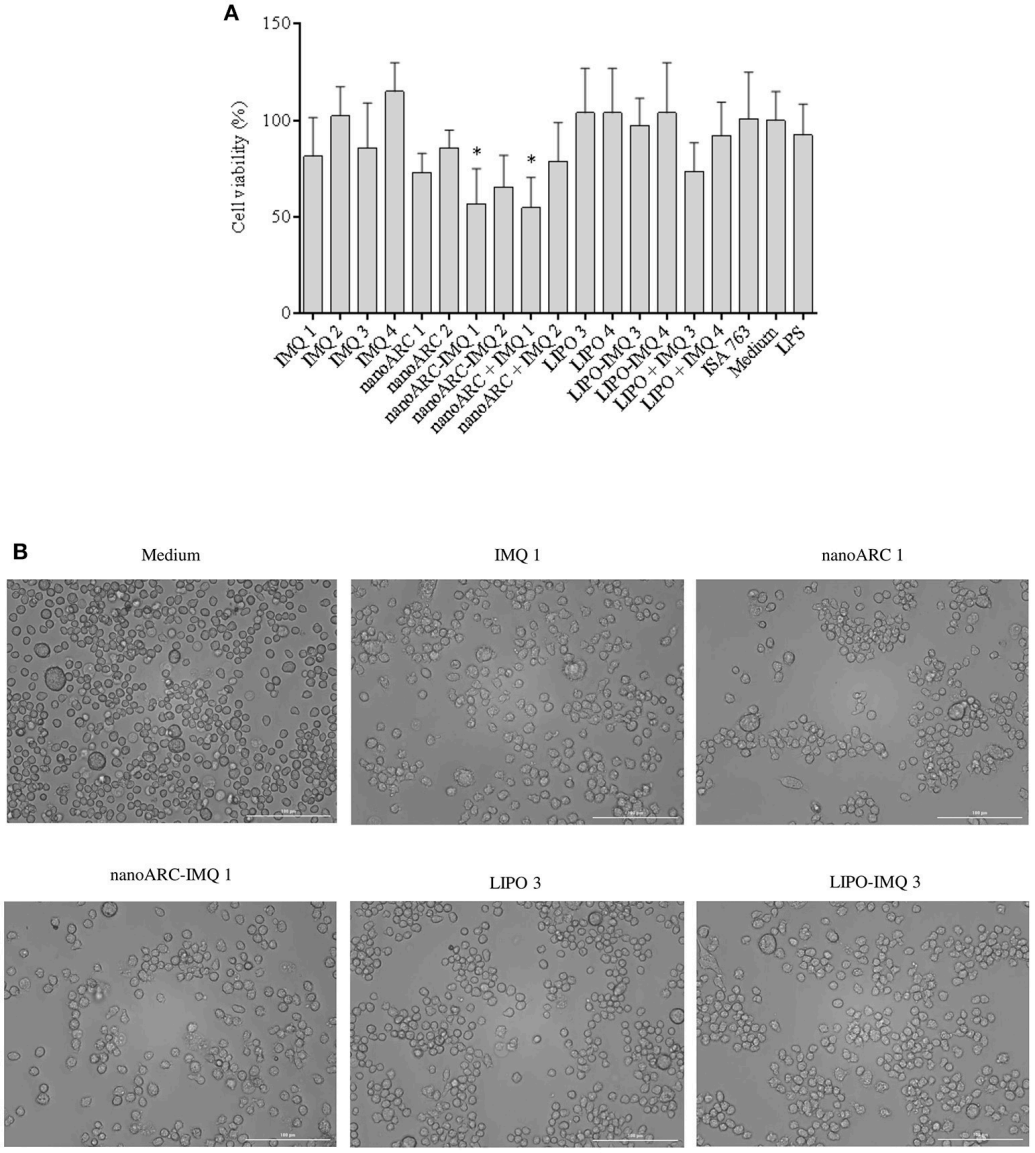
**(A)** Cell viability. J774A.1 cell viability upon 24 h incubation with free IMQ, nanoARC-IMQ and remainder IMQ formulations evaluated by MTT. Values are expressed as mean ± SD (*n* = 3 batches). Asterisks represent statistical difference against medium **(B)** bright field microscopy of selected samples, 20X magnification.

In Figure 4A the cytotoxicity of nanoARC-IMQ, LIPO-IMQ, ISA 763, and IMQ upon 24 h incubation on the TLR7 (De Meyer et al., 2012) SR-A1 (Todt et al., 2008) expressing macrophage cell line J774A.1, is shown. The cell viability was not affected between 0.5 and 5 μg IMQ, 10-100 μg PL LIPO and LIPO-IMQ. Five micrograms IMQ/100 μg PL for nanoARC-IMQ, and 100 μg nanoARC + 5 μg IMQ on the other hand, resulted relatively toxic, reducing the cell viability by ≈40%.

In Figure 4B is shown that, in line with the cytotoxicity assay and independently of its IMQ content, the nanoARC treatment reduced the relative number of cells adhered to substrate, but did not induce significant morphological alterations. The cells treated with LIPO on the other hand, seemed to suffer less drastic changes.

**Figure 5:**
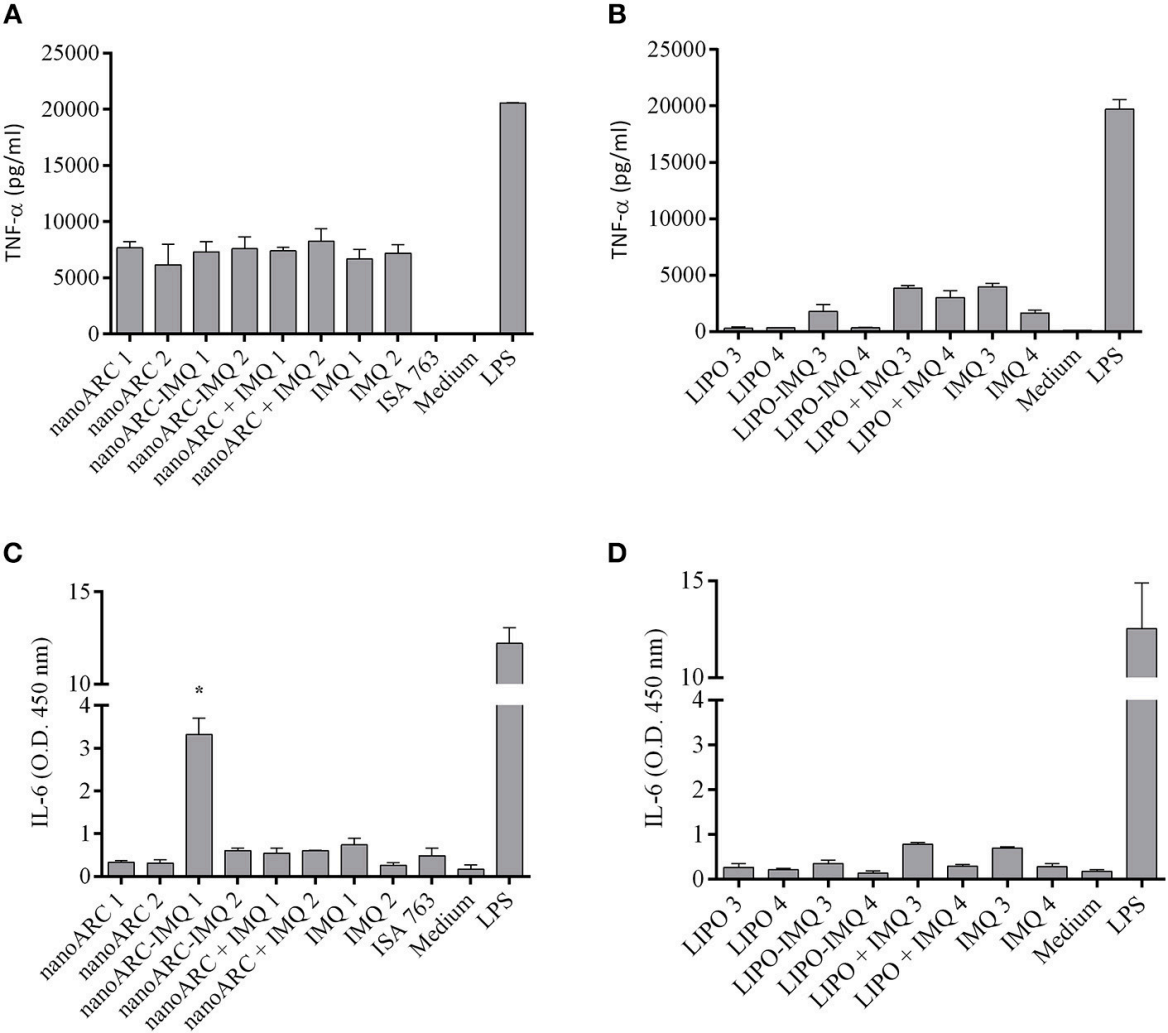
Pro-inflammatory cytokines released by J774A.1 cells. **(A,B)** TNF-α and **(C,D)** IL-6 released upon 24 h of incubation free IMQ, nanoARC-IMQ and remainder IMQ formulations. Values are expressed as mean ± SD (*n* = 3 batches). Asterisks denotes significant differences against IMQ.

NanoARC-IMQ, an internalizable SR-A1 ligand combined with a TLR7 ligand, was expected to increase the production of TNF-α and of IL-6 above that induced by IMQ or nanoARC. However, at all the tested concentrations, nanoARC-IMQ, IMQ and nanoARC induced comparable TNF-α titers (Figure 5A). 0.25 μg IMQ/100 μg PL LIPO-IMQ on the other hand, induced even less TNF-α than 0.25 μg/ml IMQ (Figure 5B). Remarkably, 5 μg IMQ/100 μg PL nanoARC-IMQ was the only formulation that increased the IL-6 titer nearly 4 folds above IMQ (Figures 5C,D).

### DTH

**Figure 6:**
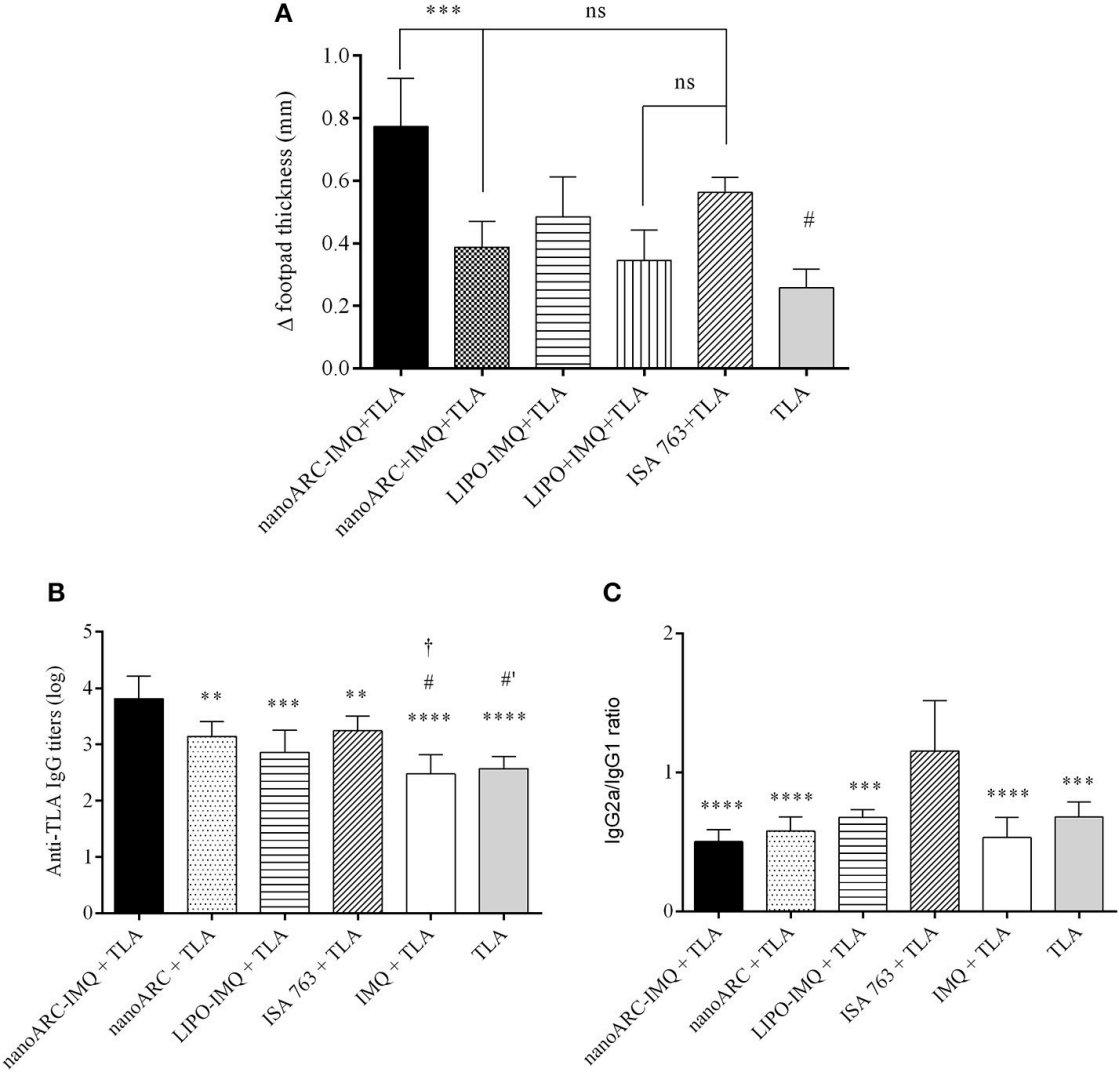
**(A)** DTH. Asterisks represent statistical difference between nanoARC-IMQ + TLA and nanoARC + IMQ + TLA and numeral represents statistical difference between nanoARC-IMQ + TLA and IMQ + TLA (#*p* < 0.01). ns, no significant difference Values are expressed as mean ± SD (*n* = 3), **(B)** Serum anti-TLA IgG titers at day 38 after 3 subcutaneous doses. Asterisks indicate significant differences between nanoARC-IMQ + TLA and remainder groups. Numerals indicate significate differences between ISA 763+TLA and IMQ + TLA or TLA (^#^*p* < 0.05; ^#^′*p* < 0.01). The cross indicates significant differences between nanoARC+TLA and IMQ + TLA (^†^*p* < 0.05). Values are expressed as mean ± SD (*n* = 3), **(C)** IgG2a/IgG1 isotype ratio at day 38. Asterisks indicate significant differences between ISA 763 + TLA and the remainder groups. Values are expressed as mean ± SD (*n* = 3).

In Figure 6A the swelling diameters induced upon 3 sc doses of nanovesicles are shown. It was observed that nanoARC-IMQ+TLA and ISA763+TLA induced equally pronounced swelling or cell mediated local responses, higher than those of the reminder IMQ formulations. Remarkably, the response to nanoARC-IMQ+TLA surpassed those induced by free IMQ+TLA and by nanoARC+ IMQ + TLA. This led us to confirm that nanoARC-IMQ, and not the free IMQ alone or mixed with nanoARC, improved the response to IMQ, a fact that resulted in a higher DTH.

Opposingly, no differences in swelling diameter were observed between LIPO-IMQ+TLA and LIPO+IMQ+TLA. The low IMQ/PL ratio, much less extensive endocytic uptake of LIPO compared to ARC (Altube et al., 2016), together with the almost absent capacity of stimulating TNF-α and IL-6 on J774A.1 macrophages that rendered a less pronounced DTH on the other hand, suggested that only ARC but not LIPO, would succeed in modifying IMQ pharmacodynamics on target cells. Despite of not all immunogens inducing DTH are associated to a protective cell response (Nichols et al., 2002), a classical DTH reaction is considered indicative of cellular protective response (Crowle, 1975), mediated by T helper 1 (Th1) cells or activated CD4+ T cells (Cher and Mosmann, 1987) and CD8+ cytotoxic T cells (Kalish and Askenase, 1999).

### Antigen-specific systemic IgG titers and IgG isotypes

In Figure 6B the antigen-specific systemic IgG titers upon 3 sc doses of nanovesicles are shown. Clearly, nanoARC-IMQ+TLA induced the highest response. Remarkably, IMQ+TLA and TLA alone induced a response ~1.5 orders lower. No difference was detected between IMQ+TLA and TLA, meaning that IMQ was useless to raise systemic antibodies. Notably (despite of administered by a different route) Aldara does not raise CD4+ T cells or antibody responses either (Rechtsteiner et al., 2005; Gogoll et al., 2012, 2016). ISA763+TLA on the other hand, raised a less pronounced response. Our results differ from those reported in Cargnelutti et al. (2016), that 36 days after 3 sc 10 folds higher TLA dose/ Balb/C mice, found no significant differences between responses to TLA and to the TLR7/8 ligand, while ISA763+TLA doubled it. In Figure 6C the IgG2a/IgG1 isotype ratio (indicative of Th1/Th2 polarization in mice (Coffman et al., 1988) is shown. The isotype ratio was ~1 for ISA763+TLA and ~0.5 for the remainder samples, meaning that whereas ISA 763+TLA recalled a mixed response, nanoARC-IMQ+TLA and the reminder groups would induce a humoral-biased response. The more detailed studies described below were aimed to compare nanoARC-IMQ with ISA763.

### IFNγ in splenocytes supernatants and intracellular IL-10 and IL-12 cytokines

**Figure 7:**
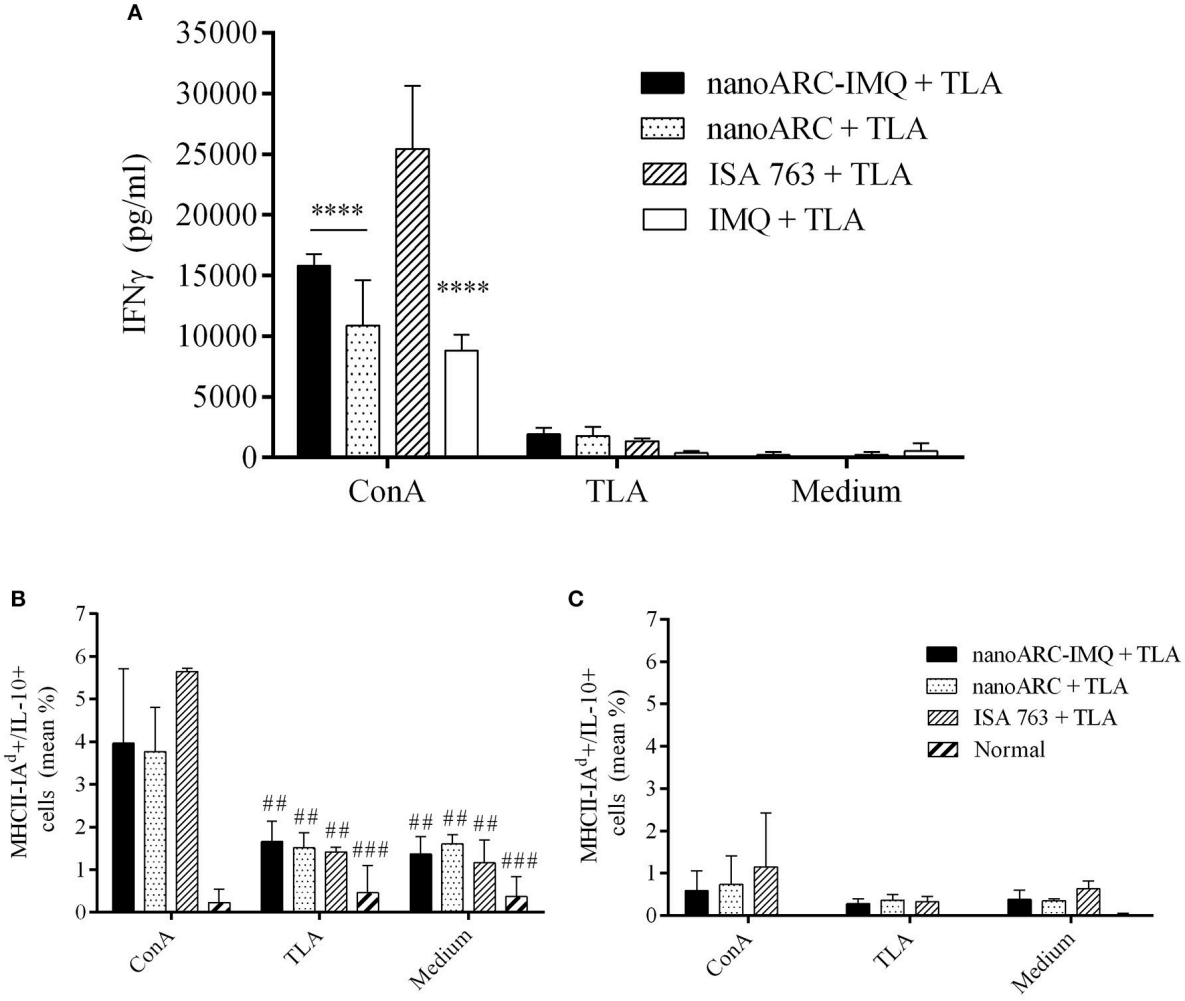
**(A)** IFN-γ production and **(B,C)** cytoplasmic antibodies labeling IL-10 cytokines of splenocytes after 48 h stimulation with concanavalin A, TLA or culture medium. **(B,C)** correspond to percentage media of activated cell [MHCII(I-A^d^)+] expressing IL-10 in the **(B)** monocytes and DCs and in **(C)** the lymphocytes regions, respectively. Asterisks indicate significant differences between ISA 763 + TLA and the reminder groups. Numerals indicate significant differences between ConA and TLA or medium. Values are expressed as mean ± SD (*n* = 3 mice per group).

In Figure 7A the production of IFNγ in splenocytes supernatant from mice immunized with nanoARC-IMQ+TLA, ARC+TLA, and ISA763+TLA, is shown. It was observed that all groups equally responded to TLA stimulus inducing low IFNγ levels (compared to those raised by concanavalin A), suggesting a mild induction of Th1 effectors in all groups. Additionally, only in the monocytes and DC region a mild nonspecific production of IL-10 was observed (Figure 7B). Neither IL-10 (Figure 7C) not IL-12 induction was observed in the lymphocytes region (data not shown).

### Splenocytes surface markers

**Figure 8:**
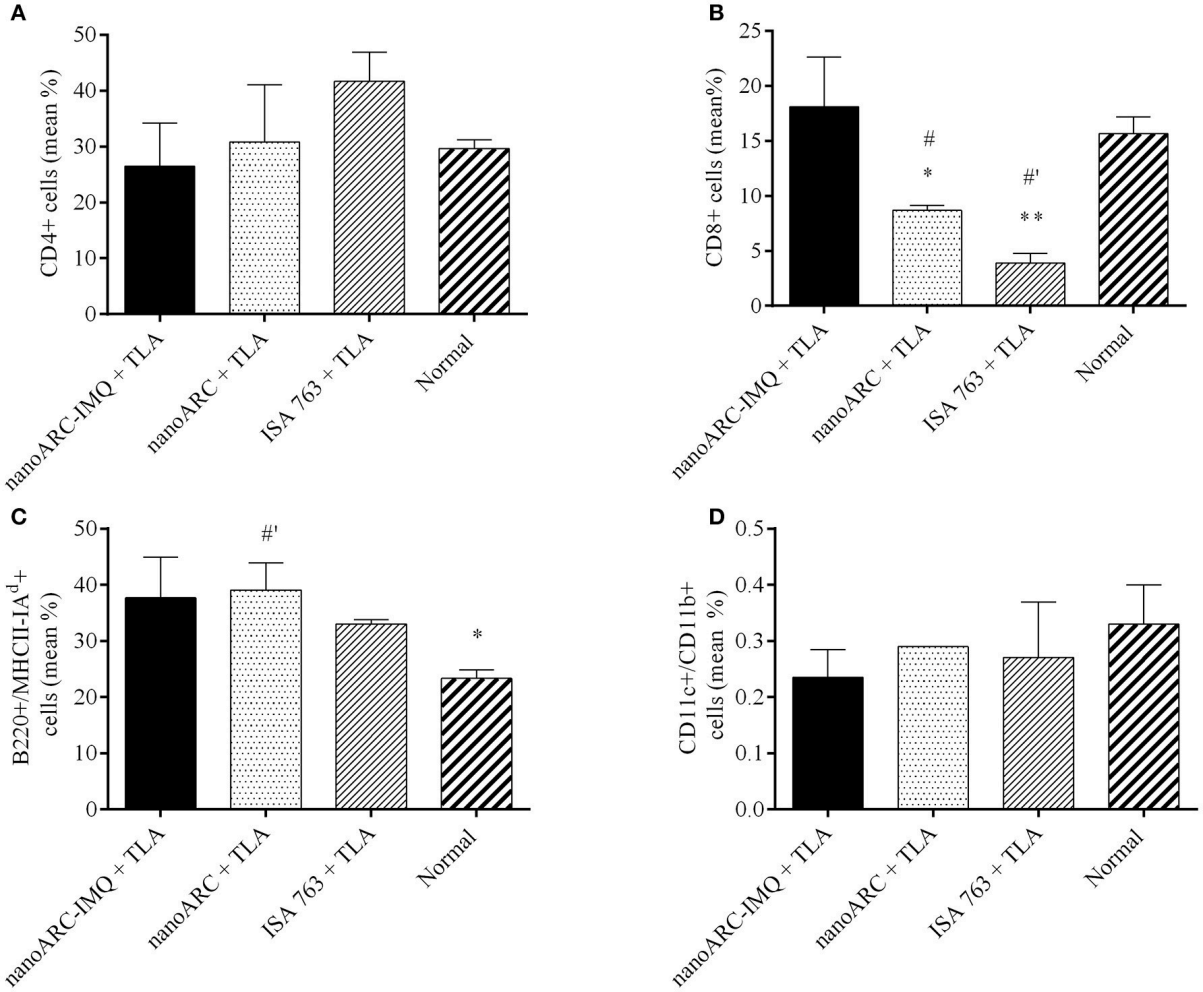
Surface cell markers. Non-stimulated splenocytes from nanoARC-IMQ+TLA, nanoARC+TLA and ISA+TLA vaccinated mice were used to assess the phenotypical expression of cell markers. **(A)** CD4 T Lymphocytes. **(B)** CD8 T Lymphocytes. **(C)** Activated B Lymphocytes (B220+/MHCII(I-A^d^)+). **(D)** CD CD11c+/CD11b+. The graphic shows the percentage average of positive cells for each marker. Asterisks indicate significant differences between nanoARC-IMQ+TLA and the remainder groups. Numerals indicate significant differences between non-immunized and the reminder groups (^#^*p* < 0.05; ^#^′*p* < 0.01).

As shown in Figure 8, despite of the mild IFNγ levels reported above, no significant increase of CD4+ TL (A) markers or CD11c+/CD11b+, marker of myeloid or classical dendritic cells (D) was induced in any group. All groups showed a non-specific increased expression of B220+/MHCII(I-Ad)+ (C), markers of mature activated B lymphocytes expressing surface MHC class II, capable of antigen processing and presentation to T cells (Hoffman et al., 2016). However, ISA763+TLA and ARC+TLA induced a pronounced decrease in CD8+ TL markers; only the group immunized with nanoARC-IMQ+TLA maintained the percentage of CD8+ TL cells at a slightly higher (tough not statistically significant) than the control value.

### Lymphoproliferation of splenocytes

**Figure 9:**
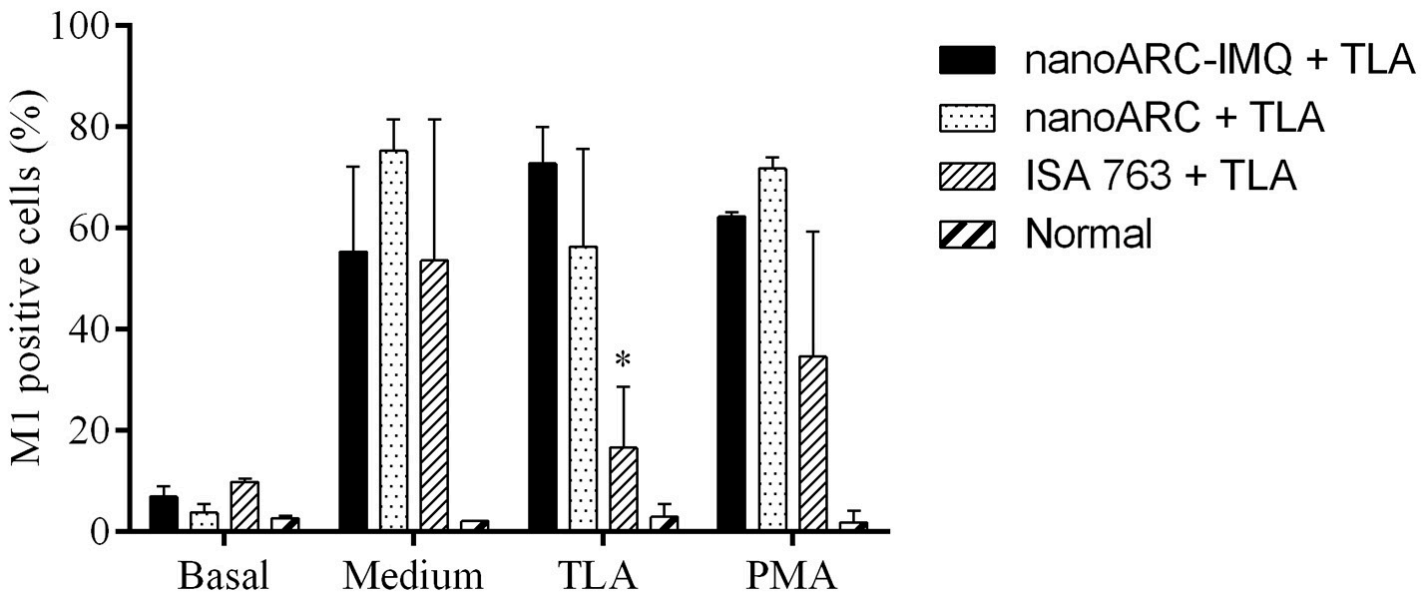
Lymphoproliferation assay. Each sample was examined by triplicate and each bar is the media ± SD of 2 mice per group. Asterisk indicates significant differences between cells stimulated respect to the control with culture media.

As shown in Figure 9, after 48 h the splenocytes experienced non-specific proliferation (as observed in culture media incubation, in the absence of TLA or PMA stimulus). The group immunized with ISA763+TLA and stimulated with TLA, however, experienced a lower lymphoproliferation than those immunized with nanoARC-IMQ+TLA or nano-ARC+TLA.

## Discussion

The presence of perpendicular methyl groups in isopranyl chains makes the archaeolipids bilayers packed loosely in comparison with bilayers made of ordinary phospholipids (Yamauchi et al., 2000). The lateral entanglement of methyl groups and the bulky carbohydrate moieties from sugar headgroups connected by hydrogen bonds (Róg et al., 2007), make the archaeolipids bilayers highly disordered and also of low lateral mobility (Kitano et al., 2003). Each archaebacteria gender on the other hand, possesses a set of characteristic archaeolipids (Corcelli and Lobasso, 2006). Specifically, the polar archaeolipids extracted from *Halorubrum tebenquichense*, were identified by ESI-MS by Higa et al. (2012) and ordered according to decrescent abundance as: archaeol analog methyl ester of phosphatidylglycerophosphate (PGPMe), archaeol analog phosphatidylglycerol (PG), (1-O-[α-D-mannose-(2′-SO3H)-(1′ α 2′)-α-D-glucose]-2,3-di-O-phytanyl-sn-glycerol) (SDGD5) the cardiolipin bis phosphatidylglycerol (BPG) and the glycocardiolipin SDGD5PA (2′-SO3H)-Manp-α1,2Glcpα-1-1-[sn-2,3-di-Ophytanylglycerol]-6-[phospho-sn-2,3-di-O-phytanylglycerol]. Recently the double negatively charged PGPMe, majoritarian polar lipid in *H. tebenquichense* responsible for its high negative Z potential of −40 mV, was confirmed to be a ligand of SRA-1 and not of SRB (the phosphatidylcholine receptor) (Altube et al., 2016).

Scavenger receptors are defined as “cell surface receptors that typically bind multiple ligands and promote the removal of non-self or altered-self targets” (Prabhudas et al., 2014). SR-A1 is a scavenger receptor of expression mainly confined to macrophages (Naito et al., 1991, 1992; Hughes et al., 1995), with unusually broad ligand binding properties including diverse types of negatively charged proteins, lipoproteins, nucleic acids, and polysaccharides (Goldstein et al., 1979 and Plüddemann et al., 2007). Remarkably, depending on the chemical nature of internalized ligand and on the microenvironment, the SR-A1 mediates either inflammatory (by secreting a spectra of pro-inflammatory cytokines) or counteracting proinflammatory receptors (Cotena et al., 2004).

Ligands internalization mediated by SR-A1 lacks- unless in case of stressed ER-, of negative feedback, being typically highly extensive (de Winther et al., 2000). Because of its content on SR-A1 ligand PGP-Me, nanovesicles and nanoparticles containing polar archaeolipids from *H. tebenquichense* experience extensive internalization as well, compared to the less pronounced of ordinary liposomes (Altube et al., 2016; Higa et al., 2017). Overall, the presence of these archaeolipids into otherwise labile nanovesicles not only avoids cumbersome chemical derivatization required for ligands linkage but provides nanovesicles with a higher tolerance to physicochemical stress (heat sterilization and storage under cold-free conditions, chemoenzymatic attacks (Caimi et al., 2017; Higa et al., 2017) than ordinary liposomes, constituting also an efficient mean to increase the intracellular delivery of loaded cargo.

In this scenario, the idea of employing SR-A1-targeted nanoARC carrying IMQ as immunomodulatory adjuvant, has two important implications. The most obvious is counting on a soft carrier, resistant to physicochemical and enzymatic attacks, to which bilayers the IMQ was strongly associated. Such interaction would reduce the chances of release and spreading of free IMQ before target cells internalization, as occurs with the current (particulate or not) IMQ formulations. The extensively internalized nanoARC-IMQ leads to an extensive endosomal delivery of IMQ to its TLR7. The other, most difficult to assess, results from the endocytic processing of nanoARC-IMQ by the complex indirect signaling route mediated by SR-A1. Overall, in a first approach, the outcome of delivering the IMQ to macrophage endosomes via SR-A1, would result in a modification of IMQ cytotoxicity and cytokine titers elicited *in vitro* as compared to the IMQ. After that, a different *in vivo* response is expected.

Our most relevant first finding was that compared to liposomes, nanoARC bilayers resulted excellent traps for IMQ: the IMQ/PL ratio of nanoARC-IMQ was nearly 20 and 10 folds higher, respectively, than those of LIPO used in this study and DPPC:DPPG:Chol nanoliposomes reported by Fox et al. (2014). The huge IMQ/PL ratio of nanoARC-IMQ may result from the particular structural features of ARC bilayers that became more ordered upon IMQ insertion. Probably the perpendicular methyl groups of archaeolipids would account for IMQ trapping, in a fashion like the interaction between IMQ and ramified isostearic acid (ISA). ISA is a mixture of branched (mostly methyl-branched) and straight-chain isomers of C18, C16, and C14 saturated fatty acids of the general formula C17H35COOH. ISA is less susceptible to color change and oxidation than the unsaturated fatty acids and has many of the properties of stearic acid. However, it has the fluidity and solubilizing properties of oleic acid (Chollet et al., 1999) employed in the commercial formulation Aldara (Walter et al., 2013). Counting on particles of high drug/carrier ratio is important because at equal uptake rate, carriers with higher drug/carrier ratio will provide a higher drug delivery/time.

Upon 24 h, nanoARC-IMQ was found to be more cytotoxic to J774A.1 macrophages than free IMQ. Its higher toxicity would result from the massive endocytic delivery of carried IMQ: while ordinarily IMQ diffuses across the cell membrane driven by its concentration gradient, endocytosed nanoARC-IMQ would provide a faster and massive endosomal delivery of IMQ. Such higher intracellular delivery of IMQ would be responsible for increased cytokines production by nanoARC-IMQ compared to IMQ. The IMQ is known to activate macrophages and other immune cells by inducing local cytokines some of which are crucial for maturation of APCs such as IFN-α, TNF-α, IL-1α, IL-1b, IL-6, IL-8, IL-10, via MyD88-dependent signaling pathway (Eng et al., 2018) with a threshold concentration for human peripheral blood monocyte cell PBMCs included macrophages of 0.5 μg/ml (~2 μM) (Gibson et al., 1995; Testerman et al., 1995; Stanley, 2002). Besides, upon SR-A1 ligand internalization, RAW264.7 macrophages induce TNF-α, IL-6, and other proinflammatory and anti-inflammatory cytokines such as IL-10 in a pattern depending on the type of ligand (Coller and Paulnock, 2001). Here we observed that both nanoARC (a SRA1 ligand) and nanoARC-IMQ (a SR-A1 and TLR7 ligand) induced TNF-α on J774A.1 macrophages and that 5 μg IMQ/ml nanoARC-IMQ was 4–6-folds more efficient inducer of IL-6 than 5 μg IMQ/ml. On the other hand, LIPO and LIPO-IMQ were less cytotoxic than nanoARC-IMQ, but barely stimulated TNF-α and did not induce IL-6. Since LIPO are not ligand of SRA1, the TNF-α induced by LIPO-IMQ was owed to the loaded IMQ. Moreover, 10 μg ARC/ml nanoARC (without IMQ, 10 folds lower lipid concentration than LIPO-IMQ), induced TNF-α in macrophages as well. NanoARC therefore, was more efficient TNF-α inducer than LIPO-IMQ; nano-ARC-IMQ on the other hand, was more efficient IL-6 inducer than IMQ and LIPO-IMQ.

Overall, these *in vitro* results suggested that, owed to their high IMQ/PL ratio, the highly SR-A1-mediated internalized nanoARC-IMQ, magnified the effect of IMQ on TLR7 expressing macrophages. It was followed that *in vivo*, the outcome could be an improved adjuvancy compared to IMQ.

The first set of sc administrations confirmed this presumption, since nanoARC-IMQ+TLA and ISA763+TLA induced the highest DTH compared to IMQ, that alone or in other any combination induced less pronounced reactions. The second set of sc administrations showed that nanoARC-IMQ+TLA induced the highest antigen-specific systemic titers, probably because of the modified pharmacodynamics of IMQ, as proposed above.

The last section compared nanoARC-IMQ+TLA with ISA 763+TLA. The water in (metabolizable, non-mineral) oil emulsion, stabilized with mannitol, and purified oleic acid from vegetable origin ISA 763 (MONTANIDE, 2017), can stimulate both humoral and cell mediated immune responses, and is considered safer than other mineral oil based Montanides. The use of Montanides in general however, is associated with several deleterious side effects (Skinner et al., 2008; Aunsmo et al., 2009; Haugarvoll et al., 2010). *In vitro*, we found that ISA 763 was a poor inducer of TNF-α or IL-6 at the tested dose (Figures 5A,C). *In vivo*, we found that nanoARC-IMQ+TLA and ISA 763+TLA induced almost identical responses. Besides of generating similar DTH and systemic IgG titers, both stimulated low levels of IFNγ, a cytokine that controls the differentiation of naive CD4+ T cells into Th1 effectors (Alving et al., 2012) but without measurable increase of TL CD4+ cells. As expected, the isotype ratio of ISA763+TLA was ≈1 (indicative of cellular and humoral response), but the decreased CD8+ TL expression and lymphoproliferation after TLA stimulus, suggested the occurrence of late deleterious effect. On the contrary, while displaying an isotype ratio of ≈0.5 (indicative of a humoral biased response), nanoARC-IMQ+TLA neither reduced lymphoproliferation or CD8+ TL expression 10 days after the last dose; on the contrary this last was slightly increased compared to the control. These preliminary results suggest that nanoARC-IMQ impacted on the innate immunity, adding IL-6 to the TNF-α induced by IMQ on J774A.1 macrophages. The activity of nanoARC-IMQ+TLA on Balb/C mice resulted in a more intense Th2-biased response than the induced by IMQ+TLA, accompanied with a CD8+ component, without deleterious effects associated to ISA76+TLA. Further studies will assess the existence of the proposed reduced systemic bioavailability and *in vivo* adverse effects provided by nanoARC-IMQ, as well as the outcome of higher doses of nanoARC-IMQ on CD8+ TL.

## Ethics statement

This study was carried out in accordance with the recommendations of Care and Use of Laboratory Animals Institutional Committee (CICUAL). The protocol was approved by the CICUAL.

## Author contributions

FP performed archaebacteria culture, preparation, and characterization of nanovesicles, immunizations, J774A1 cell cultures, MTT cytotoxicity and TNFα, and IL-6 cytokines, data analysis. AC performed archaeolipid extraction, subcutaneous administration of nanovesicles to Balb/C mice, blood extraction and sample processing. MA performed Laurdan fluorescence anisotropy and generalized polarization assessment. DC performed extraction and processing of total leishmania antigen. MV performed lymphoproliferation assay, cell markers, Interferon γ and intracellular cytokines in splenocytes. MdF and RP performed sample preparation for cryo transmission electronic microscopies and data interpretation. MM performed data interpretation and collaborate with manuscript writing. ER project leader, data interpretation and discussion, funding owner.

### Conflict of interest statement

The authors declare that the research was conducted in the absence of any commercial or financial relationships that could be construed as a potential conflict of interest.
